# Ultrasound for Non-invasive Assessment and Monitoring of Quadriceps Muscle Thickness in Critically Ill Patients With Acute Kidney Injury

**DOI:** 10.3389/fnut.2021.622823

**Published:** 2021-04-15

**Authors:** Alice Sabatino, Umberto Maggiore, Giuseppe Regolisti, Giovanni Maria Rossi, Francesca Di Mario, Micaela Gentile, Maria Teresa Farina, Enrico Fiaccadori

**Affiliations:** ^1^UO Nefrologia, Azienda Ospedaliera- Universitaria Parma, Parma, Italy; ^2^Dipartimento di Medicina e Chirurgia, Università di Parma, Parma, Italy

**Keywords:** acute kidney injury, body composition, critical care, muscle wasting, muscle ultrasound, intensive care unit

## Abstract

**Background and aims:** Critically ill patients with acute kidney injury (AKI) undergo major muscle wasting in the first few days of ICU stay. An important concern in this clinical setting is the lack of adequate tools for routine bedside evaluation of the skeletal muscle mass, both for the determination of nutritional status at admission, and for monitoring. In this regard, the present study aims to ascertain if ultrasound (US) is able to detect changes in quadriceps muscle thickness of critically ill patients with acute kidney injury (AKI) over short periods of time.

**Methods:** This is a prospective observational study with a follow-up at 5 days. All adult patients with AKI hospitalized at the Renal ICU of the Parma University Hospital over 12 months, with a hospital stay before ICU admission no longer than 72 h, and with a planned ICU stay of at least 5 days, were eligible for the study. An experienced investigator assessed quadriceps rectus femoris and vastus intermedius thickness (QRFT and QVIT) at baseline and after 5 days of ICU stay.

**Results:** We enrolled 30 patients with 74 ± 11 years of age and APACHE II score of 22 ± 5. Muscle thickness decreased by 15 ± 13% within the first 5 days of ICU stay (*P* < 0.001 for all sites as compared to ICU admission). Patients with more severe muscle loss had lower probability of being discharged home (OR: 0.04, 95%CI: 0.00–0.74; *P* = 0.031).

**Conclusions:** In critically ill patients with AKI, bedside muscle US identifies patients with accelerated muscle wasting.

## Introduction

Critically ill patients undergo major muscle wasting in the first few days of their ICU stay (1). Clinical consequences are represented by delayed functional recovery, difficult weaning from mechanical ventilation and increased mortality risk (1).

In this clinical setting an important cause of concern is the lack of adequate tools for routine bedside evaluation of the skeletal muscle mass (2). The reference methods considered as the gold standard for the assessment of skeletal muscle, such as computed tomography (CT), magnetic resonance imaging (MRI), and dual energy X-ray absorptiometry (DEXA) are not feasible for routine evaluation and monitoring of muscle mass and body composition (2). On the other hand, currently used bedside tools, such as bioimpedance analysis (BIA) and anthropometry, are not accurate enough in critically ill patients (2), mainly due to the possible interference of fluid overload, frequently observed especially when Acute Kidney Injury (AKI) coexists. Recently, the use of ultrasound (US) for the assessment of muscle dimensions has aroused considerable interest, and its reliability and validity have been documented also in critically ill patients with AKI (3, 4). US technique seems to be poorly influenced not only by fluid overload, but also by the rapid and relevant fluid shifts typical of patients with AKI undergoing Kidney Replacement Therapy (KRT) (3).

On this premise, in the present study we aimed to assess whether US is able to detect changes in muscle thickness of patients with AKI over a short period of time.

## Materials and Methods

### Patients

This is a prospective, longitudinal (5 days) observational study, conducted in the Renal ICU of the Parma University Hospital. Procedures were held in accordance to the Helsinki declaration and informed consent was obtained from patients or their next of kin. The study was approved by the local ethics committee (Comitato Etico di Area Vasta Emilia Nord, AVEN, Prot n. 43943 −03/12/2015).

All adult patients with AKI hospitalized in the Renal ICU from 15/03/2017 to 15/03/2018, with a hospital stay before ICU admission no longer than 72 hours, and with a predictable ICU stay of at least 5 days were eligible for the study. AKI was diagnosed according to KDIGO guideline criteria (5).

Already available data on quadriceps femoris US evaluation in healthy subjects (body mass index (BMI) > 18.5 Kg/m^2^, Subjective Global Assessment (SGA) class A, absence of chronic or acute illness) (6) were used for comparison with AKI patients, both at ICU admission and after 5 days of ICU stay.

### Methods

#### US Technique

The same experienced investigator (renal dietitian) performed all of the measurements. Quadriceps rectus femoris thickness (QRFT) and quadriceps vastus intermedius thickness (QVIT) were measured by B-mode ultrasonography, wall tracking ultrasound system (Philips hd7xe) with a 7.5 MHz linear array transducer (L12-3 transducer), as previously described in detail (3). All US measurements were performed in duplicate and the average of the scores used in final analyses. The transducer was placed perpendicular to the long axis of the thigh with a large amount of gel and no pressure to avoid compression of the muscle. QRFT and QVIT were measured at the midpoint (RF Prox; VI Prox) and at the border between the lower third and upper two-thirds (RF Dist; Prox Dist) between the anterior superior iliac spine (ASIS) and the upper pole of the patella (3, 7). The right and left quadriceps values were assessed in both legs with the patient lying in a supine position with both knees extended but relaxed and toes pointing to the ceiling. The assessor was positioned on the side of the patient while performing the measurements, and was allowed to tilt the probe to obtain the best possible image, in which RF and VI would be aligned and centered. Measurements were performed directly on the US machine while obtaining the images. The vertical diameter of the muscles was measured on the inner edge of the muscle fascia. US was performed twice during ICU stay, at baseline (at ICU admission) and after 5 days since the first measurement. Muscle US took <20 min to complete the image acquisition and perform the measurements.

*Demographics, clinical data, renal function and outcome:* data were collected as per institutional routine at the time of ICU admission and during ICU stay, with special regard to demographic, body weight and height, clinical and laboratory data, renal function, acute and chronic comorbidities, severity of illness (APACHE II score), data on renal replacement therapy (RRT), length of stay and mortality.

*Outcomes:* muscle loss after 5 days.

### Statistical Analysis

Results are expressed as mean and standard deviation for continuous variables with normal distribution, or median and range for non-parametric data, and as frequencies for categorical variables. Group differences were analyzed using Student *t*-test and Mann-Whitney's *U*-test for parametric and non-parametric data, respectively to assess difference between means of the control group and the patient group. ANCOVA was used to adjust the analysis by age and sex.

We examined the difference between muscle thickness at baseline and at 5 days after admission by mixed-effects models with patients fitted as random effects, and the four-way interaction term between time and each of the three sites of measurements (RF vs. VI, Left vs. Right, Proximal vs. Distal) fitted as fixed effects. We examined the relation between baseline comorbidities and change in muscle thickness by mixed-effects ANCOVA models in which muscle thickness at 5 days after admission was included as dependent variate and baseline thickness was included as covariate, in order to adjust for the correlation between change in muscle thickness and random differences in baseline values. We examined the relation between change in muscle thickness and ICU outcome (discharge, transferal to other health care facility, death) in two steps. First, we estimated the individual change over time in muscle thickness by the best linear unbiased predictions (BLUPs) of the random slope from mixed-effects random coefficients models. Then, we fitted a multinomial logistic regression model where outcome (discharge, prolonged stay, death) was the dependent variable and the individual random slope the independent variable. Because of sparse data concerning mortality (five patients only) we did not report the findings on mortality. A two-sided *P* value of less than 0.05 was regarded as statistically significant. Stata Release 16 (StataCorp, College Station, TX, US) was used for all the analyses.

### Sample Size Calculations

No data are currently available in the literature on US evaluation and monitoring of quadriceps muscle mass in patients with AKI. In a recent study on ICU patients (8) 22 patients were enrolled in order to detect a 16% reduction in the quadriceps rectus femoris thickness after 5 days of ICU stay, with a power of 80% and a probability of type I error equal to 0.05. We enrolled 30 patients to account for possible drop-outs.

## Results

Table 1 shows the baseline characteristics of the 30 patients studied. Seventy percent (21/30) were male with a mean ± SD age of 74 ± 11 years;, and they represented a severely critically ill cohort (APACHE II was 22 ± 5). A total of 472 images were analyzed across the 30 patients. Eighty-three percent of patients (25/30) were non-surgical patients and the main admission diagnosis in the ICU was renal, followed by sepsis. On average, patients were polymorbid (2.8 ± 1.7 comorbidities per patient), hypertension being the most frequent comorbidity. As to the usual renal function, 37% (11/30) had basal eGFR values <60 ml/min/1.73 m^2^ (CKD stages 2 to 5 non dialysis). At the time of first US evaluation, all of the patients had stage 3 AKI; in 21/30 patients (70%) RRT was started as 10–12 h lasting sustained low-efficiency dialysis. Oliguria was common (67%), as was sepsis (40%). ICU mortality was 17% (5/30); hospital mortality was 30% (9/30). The median (range) length of ICU stay was 15 (4-72) days, while the length of hospital stay was 34 (7-138). C-reactive protein was 109.2 mg/dL (± 68.1).

**Table 1:** Demographic and clinical data.

**Variables**	**Patients (*n* = 30)**	**Healthy subjects (*n* = 35)**
Age	74 (10.6)	41 (10.0)^*^
Male sex (*n*, %)	21/30 (70)	15/35 (43)^*^
Body weight (Kg)	82 (13.2)	70.5 (16.6)^*^
Height (m)	1.67 (0.09)	1.70 (0.09)
BMI (Kg/m^2^)	29 (4.6)	24.3 (4.6)^*^
APACHE II	22 (5)	NA
**Main admission diagnosis (*****n*****, %)**		
—Renal	18/30 (60)	NA
—Sepsis	4/30 (14)	NA
—Respiratory	3/30 (10)	NA
—Vascular	3/30 (10)	NA
—Malignancy	1/30 (3)	NA
—Cardiac	1/30 (3)	NA
**Surgical status (*****n*****, %)**		
—Urgent	2/30 (7)	NA
—Programmed	3/30 (10)	NA
—Non-surgical	25/30 (83)	NA
**Chronic comorbidities (*****n*****, %)**		
—Hypertension	23/30 (77)	NA
—Diabetes mellitus	11/30 (37)	NA
—COPD	7/30 (23)	NA
—Ischemic cardiopathy	7/30 (23)	NA
—Heart failure	8/30 (27)	NA
—Peripheral vascular disease	5/30 (17)	NA
—Immunocompromised	2/30 (7)	NA
—Chronic liver disease	2/30 (7)	NA
—Malignancy	6/30 (20)	NA
—Chronic kidney disease (not on dialysis)	11/30 (37)	NA
**Acute complications at first muscle US (*****n*****, %)**		NA
—Sepsis	12/30 (40)	NA
—Invasive mechanical ventilation	4/30 (13)	NA
—Non-invasive mechanical ventilation	7/30 (23)	NA
—Oliguria	20/30 (67)	NA
—Vasoactive drug need	7/30 (23)	NA
—Renal replacement therapy	21/30 (70)	NA
**ICU outcome (*****n*****, %)**		
—Death	5/30 (17)	NA
**Hospital outcome (*****n*****, %)**		
—Death	9/30 (30)	NA
—Discharged home	15/30 (50)	NA
—Transferred to long-stay/rehabilitation ward	2/30 (7)	NA
—Transferred to another hospital	4/30 (13)	NA
ICU LOS median, range)	15 (4-72)	NA
Hospital LOS (median, range)	34 (7-138)	NA
***Biochemical data***		
sCr (mg/dl)	6.3 (4)	NA
BUN (mg/dl)	81.7 (36)	NA
Albumin (g/dl)	2.8 (0.6)	NA
CRP (mg/dl)	109.2 (68.1)	NA

*Data expressed as mean (standard deviation), frequencies and median (range)*.

* *P < 0.001 in comparison to AKI patients. BMI, body mass index; BUN, blood urea nitrogen; COPD, chronic obstructive pulmonary disease; CRP, C-reactive protein; ICU, intensive care unit; IMV, invasive mechanical ventilation; LOS, length of stay; NIMV, non-invasive mechanical ventilation; sCr, serum creatinine*.

### Patients With AKI in Comparison to Healthy Subjects

**Figure 1:**
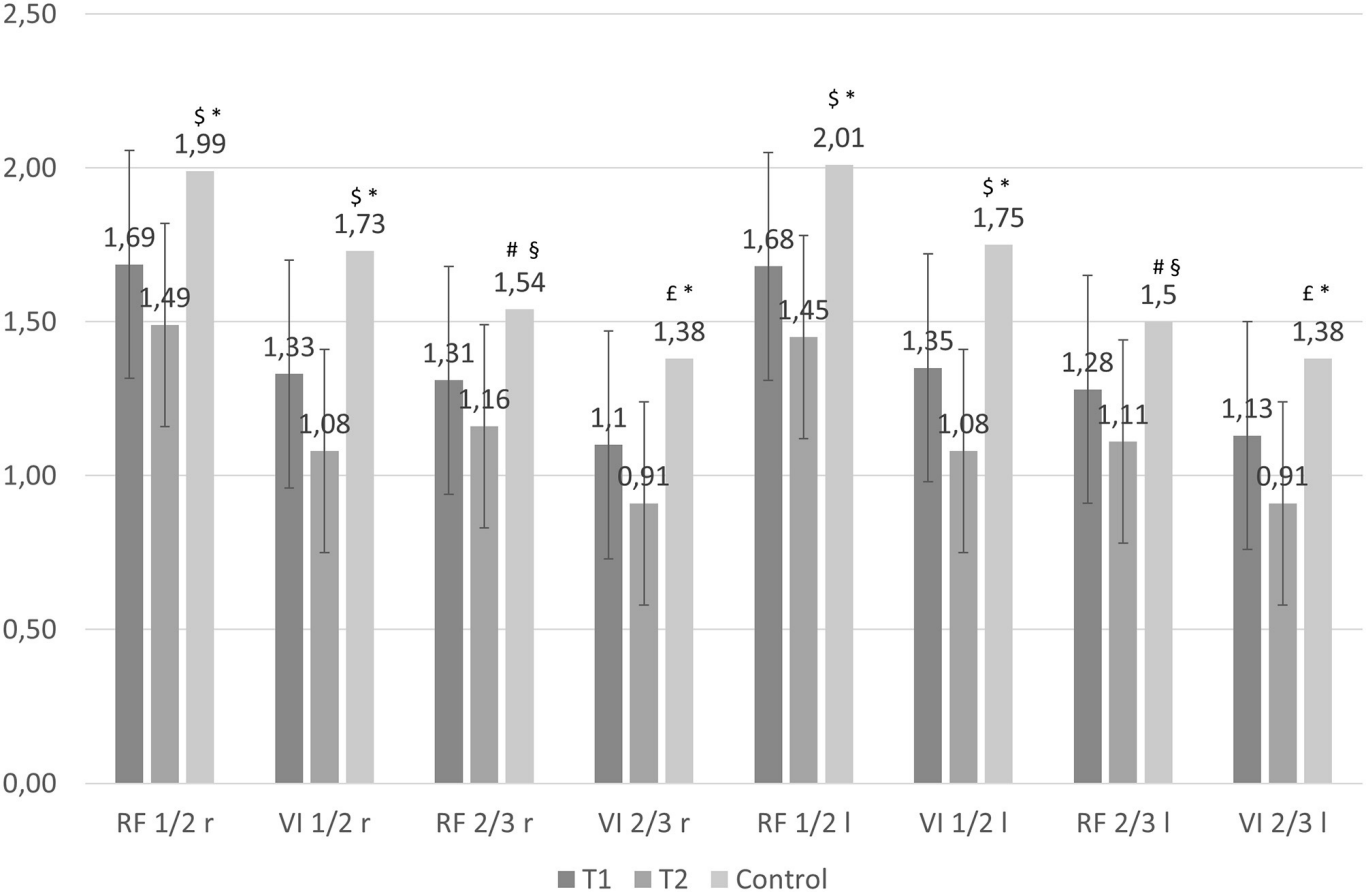
Muscle thickness of patients with AKI (baseline, T1, and after 5 days, T2), and control group. The first 2 columns represent the mean and 95 percent confidence interval (vertical bar) of muscle thickness at each measurement site as estimated by the mixed effect model, at baseline (T1) and after 5 days (T2). The third column represents the control group. The average difference between baseline and 5 days was statistically significant at all measurement sites (*P* < 0.001). When comparing the difference between patients and healthy subjects, muscle thickness was different between controls and baseline (T1) values of patients: ^$^*P* = 0.001, ^#^*P* < 0.05, *P* < 0.01; *P* < 0.001 in comparison to muscle thickness of patients after 5 days (T2). After adjusting the analysis for age and sex using ANCOVA, no statistically significant difference was found between T1 values and controls; when comparing to T2 values, muscle thickness difference remained statistically significant for all sites: *P < 0.01, ^§^*P* < 0.05.

Demographic characteristics of control group (35 healthy subjects) are shown in Table 1, while US quadriceps muscle data are illustrated in Figure 1. In general, the control group was younger and leaner than patients. At univariate analysis (Figure 1), muscle thickness of patients differed from that of the control group for all sites, both at T1 and T2. We also performed an adjusted analysis using ANCOVA corrected for age and sex. In the adjusted analysis, no difference was found between T1 values of muscle thickness and control group values; however, the difference between T2 values and the control group values remained statistically significant.

### Changes in Muscle Thickness by US

Baseline (T1) and after 5 days (T2) mean ± SD values for RF and VI thickness are illustrated in Figure 1; the difference between means was statistically significant for all sites (*P* < 0.001). On average, there was a mean clinically relevant reduction of 15% (± 12%) in every site of measurement within the first 5 days of ICU stay (Table 2). Changes in VI Prox and Dist thickness (16–19%) were greater than in RF Prox and Dist thickness (11–13%).

**Table 2:** Average percent reduction of muscle thickness after 5 days of ICU stay.

**Muscle site**	**Average reduction (SD)**
QRFT Prox r (*n* = 29)	11% (8%)
QVIT Prox r (*n* = 28)	18% (14%)
QRFT Dist r (*n* = 30)	11% (10%)
QVIT Dist r (*n* = 30)	17% (12%)
QRFT Prox l (*n* = 30)	13% (10%)
QVIT Prox l (*n* = 29)	19% (16%)
QRFT Dist l (*n* = 30)	12% (10%)
QVIT Dist l (*n* = 30)	16% (15%)
All measurements (*n* = 236)	15% (12%)

*Dist, distal; Prox, proximal; QRFT, quadriceps rectus femoris thickness; QVIT, quadriceps vastus intermedius thickness; Values expressed as mean (standard deviation)*.

**Figure 2:**
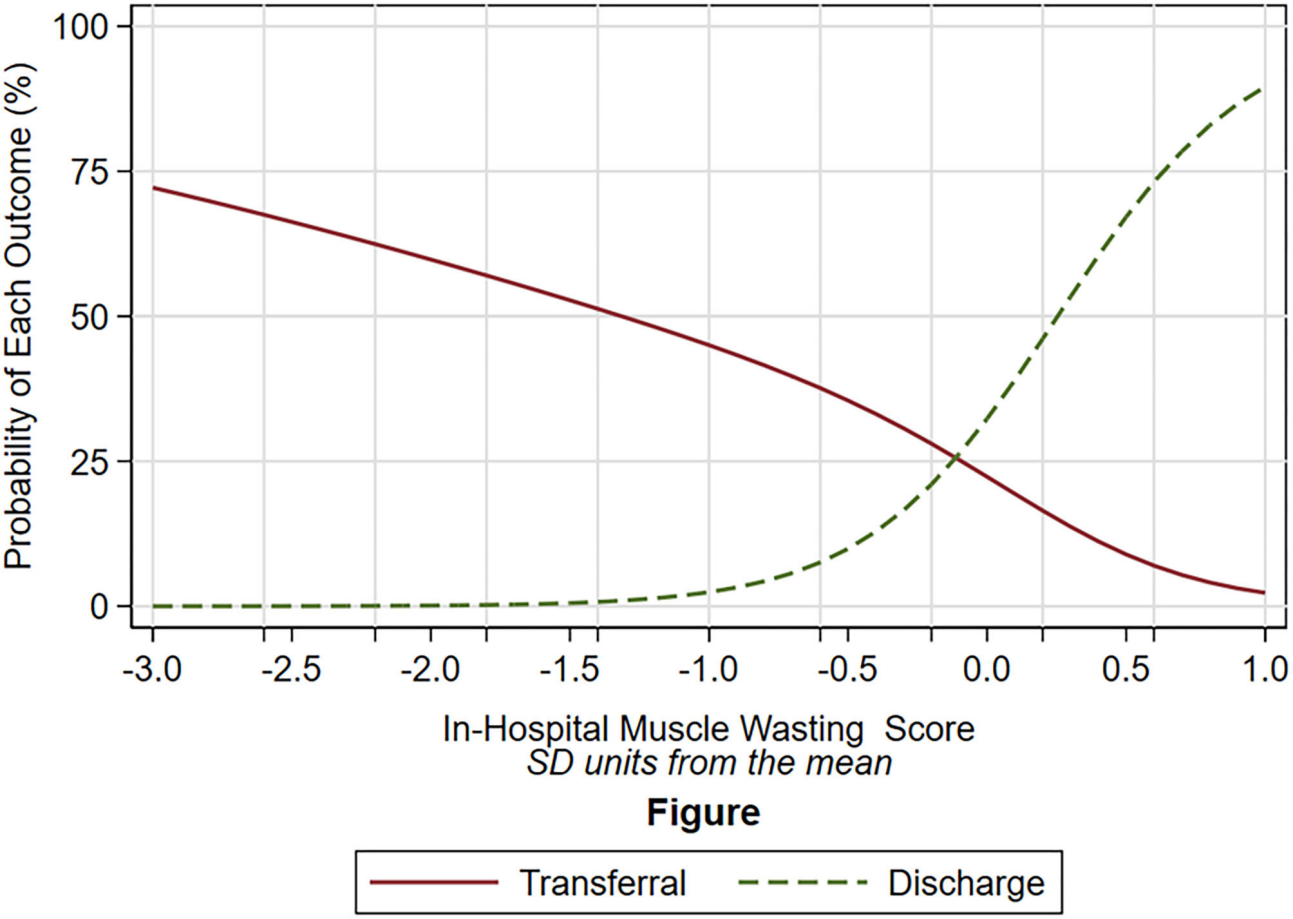
Probability of discharge (dot green line) and of transferal to rehabilitation unit (solid red line) according to the degree of muscle wasting (x-axis). The degree of muscle wasting is expressed as standard deviation unit from the mean, in which a negative number indicates higher muscle wasting, a positive number lower muscle wasting. The different shape of the relation between the dot green line and the solid red line was statistically significant (*P* = 0.031). The probability of discharge was based on multinomial logistic model (mortality is not plotted because only five patients died). The independent variable of the multinomial logistic model was the degree of muscle wasting which was estimated by the mixed models (see text).

We did not find any association between baseline muscle thickness, inflammatory status (assessed by CRP and serum albumin), as well as chronic and acute comorbidities, with muscle loss (Supplementary Table 1). Patients with the more severe muscle loss had a higher probability of having a prolonged hospital stay, which was determined by being transferred to another hospital instead of discharged home (OR: 0.04, 95%CI: 0.00- 0.74; *P* = 0.031) (Figure 2).

## Discussion

Our study confirms that muscle loss may occur early and rapidly in the first 5 days of ICU stay, and extends previous observations in general ICU patients also to patients with AKI.

At baseline, values of muscle thickness were not different from those of younger healthy subjects when the analysis were adjusted for age and sex. They were also similar to values previously reported in another study assessing elderly healthy subjects (9). However, at day 5, muscle mass was significantly reduced, both in comparison to baseline values and in comparison to the control group.

In our study, the amount of muscle loss was similar to that observed in critically ill patients (8); in particular, at day 5, the VI muscle had the most important reduction in comparison to the RF muscle. Rectus femoris is often described as a power muscle designed to assist in fast movements, while VI is considered a stabilizing muscle that is important for maintaining posture. The identification of which muscles are more affected by immobilization and critical illness in ICU patients could provide important indications in order to more precisely target rehabilitation according to the type of muscle predominantly affected (VI-postural or RF-power).

We demonstrated that an inverse relationship exists between the severity of muscle loss and the probability of discharge home. Although we can't ascertain the causal relationship between muscle loss and outcome due to the small sample size, baseline sarcopenia assessed by US predicts adverse discharge disposition (death/transferal to nursing facilities) (10). In addition, muscle loss during ICU stay is a major contributor to functional disability (11). However, despite the rapidly loss faced during the first week of ICU stay, it remains unclear whether it is the change in muscle size from baseline or the total amount of muscle mass at admission that is the most important predictor of functional outcome and mortality (12). In our study, baseline muscle thickness did not correlate to the amount of muscle loss, which is in accordance to previous studies investigating muscle wasting in critically ill patients (11).

Despite its increasingly recognized importance, the assessment of muscle mass is challenging in the ICU setting, especially when inflammation and fluid imbalance are present. Currently available bedside methods (such as anthropometry and bioimpedance spectroscopy [BIS]) have important intrinsic limitations, due for example to fluid shifts typical of critically ill patients with AKI (13). Current recommended reference methods are hardly feasible in this clinical setting. Dual energy X-Ray absorptiometry (DEXA), which has been used and recommended by recent Consensus on sarcopenia to assess appendicular skeletal muscle mass (14), is also influenced by hydration status, because it assumes that lean body mass has a constant hydration of 73%, which is not the case in critically ill patients with AKI (15). In addition, DEXA is not feasible at the bedside and involves patient radiation exposure. Computed tomography (CT) has been recently used to assess muscle mass and its correlation with mortality in critically ill patients (16). Despite its excellent accuracy, CT is expensive, requires specialized personnel, and is available for muscle mass assessment only when CT is necessary for other diagnostic procedures on the lung or the abdomen. In recent years, muscle US has been increasingly studied in the kidney patient setting. Specifically, the reliability of the muscle US technique applied in the present study has been already reported in critically ill patients with AKI, along with excellent intraclass correlation coefficient (ICC) for inter and intra-operator comparisons (3). This methodology has already been validated in patients with AKI against muscle CT (4). Despite the lack of reference values to be applied at baseline and identify patients with pre-ICU low muscle mass and, therefore, increased nutritional risk, in the present study we showed that quadriceps muscle US is useful to monitor nutritional status of critically ill patients with AKI, and that increased muscle loss reduces the chance of being discharged home.

The main limitation of the present study relies in the small sample size, that does not allow further analysis regarding the effect of muscle loss and baseline muscle assets on mortality or functional outcomes. Further studies will be needed on larger cohorts of patients with AKI to allow for such analyses.

In conclusion, muscle wasting occurs early and rapidly within the first 5 days of ICU stay in critically ill patients with AKI. Muscle US is a sensible and feasible tool for the detection of muscle wasting even in this clinical setting; moreover, it is easy to use, cheap and time-efficient. In the future, studies defining cut-off values for muscularity are needed to allow the early identification of patients with low muscle mass at ICU admission.

## Data Availability Statement

The raw data supporting the conclusions of this article will be made available by the authors, without undue reservation.

## Ethics Statement

The studies involving human participants were reviewed and approved by Comitato Etico di Area Vasta Emilia Nord, AVEN, Prot n. 43943 −03/12/2015. The patients/participants provided their written informed consent to participate in this study.

## Author Contributions

AS: investigation, methodology, validation, and writing–original draft. UM: formal analysis, writing–review and editing. GR, GMR, MG, and MF: writing–review and editing. FD: resources, writing–review and editing. EF: supervision, writing–review and editing. All authors contributed to the article and approved the submitted version.

## Conflict of Interest

The authors declare that the research was conducted in the absence of any commercial or financial relationships that could be construed as a potential conflict of interest.
