# Supplementary material for: Ultrasound for Non-invasive Assessment and Monitoring of Quadriceps Muscle Thickness in Critically Ill Patients With Acute Kidney Injury
Source: Front Nutr. 2021 Apr 15;8:622823. doi: 10.3389/fnut.2021.622823 (PMC8081900; doi:10.3389/fnut.2021.622823)
Supplement: Supplementary file 1 [file Table_1.DOCX]

**Supplementary table 1.** Relation between baseline chronic and acute comorbidities and change in muscle thickness (ANCOVA).

|  | **Coefficient (SE)** | **95% Confidence interval, P** |
| --- | --- | --- |
| ***Chronic comorbidities*** | | |
| Hypertension | 0.132 (0.13) | -0.25; 0.37, 0.692 |
| Diabetes | -0.012 (0.20) | -0.40; 0.38, 0.954 |
| COPD | 0.041 (0.33) | -0.61; 0.69, 0.899 |
| CAD | 0.026 (0.13) | -0.19; 0.25, 0.814 |
| Heart failure | -0.141 (0.10) | -0.33; 0.05, 0.154 |
| Peripheral vascular disease | 0.132 (0.17) | -0.20; 0.46, 0.436 |
| Immunocompromised | -0.002 (0.37) | -0.73; 0.72, 0.995 |
| Liver disease | 0.103 (0.44) | -0.76; 0.97, 0.815 |
| Malignancy | 0.009 (0.14) | -0.27; 0.29, 0.945 |
| CKD | 0.112 (0.13) | -0.14; 0.37, 0.389 |
| ***Acute comorbidities*** | | |
| Sepsis | 0.007 (0.18) | -0.34; 0.35, 0.967 |
| IMV | 0.028 (0.24) | -0.44; 0.50, 0.907 |
| NIMV | -0.029 (0.16) | -0.34; 0.29, 0.857 |
| Oliguria | 0.067 (0.10) | -0.13; 0.26, 0.508 |
| Vasoactive drugs | 0.180 (0.24) | -0.28; 0.64, 0.443 |
| RRT | -0.110 (0.23) | -0.28; 0.64, 0.443 |
| Shock | -0.087 (0.44) | -0.96; 0.78, 0.844 |
| Major bleeding | 0.167 (0.48) | -0.78; 1.11, 0.729 |
| APACHE II | 0.004 (0.02) | -0.02; 0.03, 0.795 |
| ***Biochemical markers*** | | |
| CRP | -0.007 (0.00) | -0.003; 0.002, 0.564 |
| Serum albumin | 0.100 (0.12) | -0.14; 0.34, 0.406 |

Values expressed as mean (± SE). CAD, coronary artery disease; COPD, chronic obstructive pulmonary disease; CKD, chronic kidney disease; CRP, C-reactive protein; IMV, invasive mechanical ventilation; NIMV, non-invasive mechanical ventilation.
